# Biocompatibility of Materials Dedicated to Non-Traumatic Surgical Instruments Correlated to the Effect of Applied Force of Working Part on the Coronary Vessel

**DOI:** 10.3390/ma18245645

**Published:** 2025-12-16

**Authors:** Marcin Dyner, Aneta Dyner, Adam Byrski, Marcin Surmiak, Magdalena Kopernik, Katarzyna Kasperkiewicz, Przemyslaw Kurtyka, Karolina Szawiraacz, Kamila Pietruszewska, Zuzanna Zajac, Lukasz Mucha, Juergen M. Lackner, Michael Berer, Boguslaw Major, Marcin Basiaga

**Affiliations:** 1 Faculty of Science and Technology, Jan Dlugosz University, 42-200 Czestochowa, Poland; 2 Chirstom Marcin i Marek Dyner S.C., 42-240 Rudniki, Poland; a.dyner@chirmed.pl; 3 Institute of Metallurgy and Materials Science, Polish Academy of Sciences, 30-059 Cracow, Poland; a.byrski@imim.pl (A.B.); pkurtyka@frk.pl (P.K.); k.szawiraacz@imim.pl (K.S.); zuzannazajac22@gmail.com (Z.Z.); b.major@imim.pl (B.M.); 4 Department of Internal Medicine, Faculty of Medicine, Jagiellonian University Medical College, 31-066 Cracow, Poland; marcin.surmiak@uj.edu.pl; 5 Center for the Development of Therapies for Civilization and Age-Related Diseases, Jagiellonian University Medical College, 31-066 Cracow, Poland; kamila.pietruszewska@uj.edu.pl; 6 AGH University of Krakow, 30-059 Cracow, Poland; kopernik@agh.edu.pl; 7 Institute of Biology, Biotechnology and Environmental Protection, Faculty of Natural Sciences, University of Silesia in Katowice, 40-032 Katowice, Poland; katarzyna.kasperkiewicz@us.edu.pl; 8 Foundation of Cardiac Surgery Development, 41-800 Zabrze, Poland; lmucha@frk.pl; 9 Institute for Sensors, Photonics and Manufacturing Technologies, JOANNEUM RESEARCH Forschungsgesellschaft mbH, 8712 Niklasdorf, Austria; juergen.lackner@joanneum.at; 10 Polymer Competence Center Leoben GmbH, 8700 Leoben, Austria; michael.berer@pccl.at; 11 Silesian University of Technology, 44-100 Gliwice, Poland; marcin.basiaga@polsl.pl

**Keywords:** atraumatic surgical instruments, biocompatible 3D printing resins, metamaterial-based medical devices, vascular compression modeling, cytotoxicity and biofilm resistance

## Abstract

Cardiovascular clamping procedures can cause tissue traumatization, leading to serious adverse events interrupting blood flow and causing life-threatening hemorrhage. The aim of the study is to evaluate the properties of 3D-printed, high-elasticity elastomeric materials—BioMed Flex 50A and 80A (Formlabs Inc., Sommerville, MA, USA)—in terms of their suitability for the fabrication of atraumatic inserts used for surgical clamping instruments. To show the importance of the elaboration of the new atraumatic materials, finite element simulations of blood vessel compression by a surgical tool were validated experimentally with porcine vessels, and histopathology assessed the tissue response. These results confirm that excessive clamping forces can cause vessel wall stratification and rupture. Specimens BioMed Flex 50A and 80A underwent surface, mechanical, and biological testing, including topography, wettability, acoustic microscopy for structural voids, cytotoxicity with human dermal fibroblasts, pro-inflammatory marker analysis, and bacterial biofilm assessment. The results of the testing of the 3D-printed BioMed Flex 50A and 80A materials show good potential for applications in safe atraumatic surgical instruments. Further research may include the possibilities to develop 3D-printed metamaterials with pressure adapting properties.

## 1. Introduction

The problem of tissue trauma during grasping in CSVS (cardiovascular and thoracic surgeries) refers to vascular clamps and laparoscopic instrument tips. The most common postoperative complications caused by current tools may cause arterial stenosis and subsequent free flap necrosis, endothelial and arterial wall damage, complete fracture into the media of the vessel, blood vessel clots interrupting blood flow to an organ or extremity, and life-threatening hemorrhage.

Pressure-matching soft materials and sensor-based solutions are most often present at the research stage, leading to less tissue trauma and increased data on how the surgeon interacts with the patient’s body. As the commonly used cardiovascular and thoracic clamping and grasping instruments are not real-time, pressure-controlled devices, almost all vascular procedures bring the risk of excessive and uneven clamping force. Also, so called atraumatic materials do not provide reversible deformation properties up to allowable limits, as the minimum operational forces should not be exceeded.

Most of the short- and long-term postoperative complications are insufficiently correlated to instrument performance in clinical practice, for example in WHO (World Health Organization) or national statistics [1,2,3,4,5,6]. Vascular clamps currently have no relevant normative requirements specifying the values of pressure forces for individual models of clamps. However, the pressure required to successfully occlude the vessel during surgery may vary during surgery as the hemodynamic parameters and vessel structure may differ from patient to patient. Problems of uneven pressing force have been partly solved by Kowalski clamps [7,8], where an even pressure force is the result of properly designed jaws ensuring their even descent. This solution has been adapted for large hinge clamps [9,10]. Clamp manufacturers additionally use various types of atraumatic serration (Model De Bakey) on the working parts of the tools, also ensuring adequate traction. However, if they are made of stainless steel or titanium, the clinical data shows their insufficient atraumatic action. Applied Medical offers replaceable atraumatic inserts [11,12] made of various materials (non-latex: Latis, Fibra, Soft, and Traction) [13,14] and Stealth forceps with silicone foam inserts with nylon fibers on the jaw surface [15,16]. Studies confirm that arterial clamps maintain function and integrity of endothelial cells [17,18]. Fogarty clamps from Applied Medical are described to possess a mostly homogeneous distribution of the clamping forces [19,20].

Metamaterials can behave as overload protection [21]. To avoid tissue damage during surgeries, only minimal operational forces should be used on tissues during grasping and clamping. Currently, only geometrical regulators for excessive forces are used (stop pins, atraumatic inserts, special atraumatic serration, and shape of the jaws). The key issue will be addressed by metamaterials, which are engineering materials possessing properties that are not found in naturally occurring materials, with increased reversible, spring-like deformation potential, whereby the deformation is controllable by the used lattice structure and even enables a fully reversible cyclic deformation with “ideal-elastic–ideal-plastic” behavior for metals (by chiral and anti-chiral materials). For such an application of metamaterials, example shown potentials are (i) shape-matching features for the design of soft grippers for touching delicate objects with the maximum surface contact and, thus, minimum contact force [22,23], (ii) shape-shifting features, (iii) biomimetic mechanical metamaterial and biomimetic multifunctional structures, etc. [24,25]. There is no other data available about the application of metamaterials for atraumatic effect.

Although the general concept of chiral and anti-chiral metamaterials is present in the literature, most of these studies were conducted on polymer structures with dimensions of the order of several centimeters.

Rigidity of a structure or material is a key concept in product design. Materials which have adjustable mechanical properties and adaptable systems are necessary. Such properties may differ greatly depending on a particular structure, and the capacity of such property designs can lead to alternative design strategies. In materials science, the application of external stimuli is employed, such as temperature in shape-memory polymers (SMPs) [1,2,3,4], magnetic fields [5,6], and electric fields [9,10]. These are the means of tunable mechanical behavior. The range of variation is therefore limited and often occurs in discrete steps (rigidity switching [11,12,13,14,15,16]). Another possibility for achieving tunable mechanical properties is metamaterials. In this respect, it is a topic of considerable interest to structural engineers [17,18,26,27]. Mechanical metamaterials are derived from geometry rather than composition, and thus have a unique mechanical behavior. Among these are lightweight structures (LSs) and (anti-/meta-) chiral structures (AMCs), which have attracted considerable scientific interest [22,23,24,25,28,29,30,31,32]. The challenge and main innovation in the work is to apply this feature to structures with significantly smaller dimensions. The change from polymer to metal changes the behavior of the parent material significantly (viscoelastic vs. elastic–plastic material behavior). Additionally, the comparatively small dimensions required for the clamps pose further challenges to the additive manufacturing (AM) by powder bed fusion (PBF). For PBF-manufactured parts, the surface roughness and the bulk porosity (coming from the powder particles), surface waviness (due to the layer-by-layer buildup) and residual stresses (due to the fast cooling) are important part property limitations.

The other problem is hospital-acquired infections and growing antibiotic resistance of bacteria, remaining a significant challenge for modern medicine [33,34,35,36,37,38,39]. One approach to minimize the risk of fatal infection is to use bacteriostatic coatings on surgical instruments. The work concerns the microbiological assessment of the risk of bacterial biofilm formation on materials intended for tools dedicated to blood vessel surgery, particularly the flow closure clamp. The core area of our innovations is cardiovascular surgery. State-of-the-art cardiovascular and thoracic clamping and gripping instruments (CVTCGI) are not devices with real-time pressure force control. Simultaneously, the work documents tissue traumatization, leading to serious adverse events, interrupting blood flow and life-threatening hemorrhage. A correlative analysis of the interaction between cells, bacteria, and the surface of a material intended for surgical instruments was considered.

## 2. Materials and Methods

### 2.1. Material Elaboration

Notwithstanding the mounting evidence of the benefits of 3D-printed medical models, instruments, and devices, many healthcare professionals remain hesitant to fully incorporate these models, devices, and resources into clinical processes. This caution is not unreasonable—patient outcomes are at stake, and every part should meet the best possible safety and performance standards. Several 3D printing materials historically lacked full certification, reliability, or clinical validation. The biocompatible range of materials created by Formlabs is intended to be the best suited for a variety of healthcare applications. Examples such as BioMed Amber Resin and BioMed White Resin (Formlabs Inc., Somerville, MA, USA) are successful materials for surgical guides and custom medical tools, but multiple hospitals and device makers suggest that physicians often prefer materials with greater impact and wear resistance. In patient-specific tools or surgical devices, clinicians are very careful not to make use of fragile parts that could come apart during demanding operations. Each part in critical environments, such as operating rooms, should ensure consistency, robustness, and dependability. Furthermore, conventional resins are often stiff and do not provide adaptability in contact with vessels or tissue. Therefore, we decided to investigate two materials: BioMed Flex 50A and BioMed Flex 80A. Both are elastic and transparent materials for biomedical applications. The materials are manufactured according to ISO 13485 [40] and tested for long-term skin (>30 days) or short-term mucosal membrane contact (<24 h), demonstrating good potential for tissue-contact applications. The primary distinction between the two materials lies in their stiffness and Shore hardness. As per the datasheets, BioMed Flex 50A exhibits a tensile strength of 2.3 MPa and a Shore hardness of 50A [41]. In contrast, BioMed Flex 80A has a tensile strength of 7.2 MPa and a Shore hardness of 80A [42].

The specimens were prepared in the form of discs with diameter 14 mm and thickness 1 mm. They were printed on Formlabs Form 3B+ polymer 3D printing (Formlabs Inc., Somerville, MA, USA) machine used for additive manufacturing—Low Force Stereolithography.

### 2.2. Surface Analysis

#### 2.2.1. Topography Tests

The surface topography was analyzed using a digital microscope ZEISS Smartzoom 5 (Carl Zeiss, Oberkochen, Germany). The system enabled a macro recording mode to enhance the workflow for repeat sample analyses of the same type in a step-by-step manner.

Orientation of the samples was supported by integrated QA/QC graphic user interface supporting a seamless macro-to-detail workflow. The entire sample surface was recorded with separate optics. The surface analysis was supported by ConfoMap^®^ Surface Imaging and Analysis Software for ZEISS microscopes V10 (Carl Zeiss, Jena, Germany). This surface metrology and analysis software is specifically dedicated to confocal, digital and widefield microscopes that produce topography maps. The software includes profile roughness and waviness analysis, basic analysis of surface data, stitching of images and surface outlier removal multifocus reconstruction.

#### 2.2.2. Contact Angle

One of the key surface parameters influencing cell interaction, alongside topography, is wettability. This property impacts protein adsorption, cell adhesion, and various biological processes on biomaterial surfaces, making its analysis vital for evaluating the biocompatibility of modified materials [43,44]. The contact angle was measured for the following materials: Biomed Elastic 50A, Biomed Flex 80A (Formlabs, Somerville, MA, USA) without and after one, two, and three sterilization cycles (using ethylene oxide and autoclave) (Table 1). The contact angle was measured using an optical goniometer, Drop Shape Analyzer DSA100 (KRÜSS, Hamburg, Germany), along with DSA4 software version 2.1 for analyzing the shape of the drop deposited on the material. The measurements were performed using the sessile drop method. Distilled water and PBS were used as measurement liquids according to ASTM D7334 standard [43]. The volume of the measuring drop deposited on the material using a pipette was 0.5 µL. For each material and measurement liquid type, the measurement was performed 10 times.

#### 2.2.3. Non-Destructive Analysis of the Risk of Creating Voids in the Material in the Emergence of Acoustic Tools

The scanning acoustic microscope (SAM Evolution II, Kraemer Sonic Industries (KSI), Herborn, Germany) operates in pulse reflection mode. The most important element in scanning acoustic microscopy is a high-frequency sound wave and a piezoelectric transducer. This element transmits and receives high-penetration sound pulses. Based on this principle, maps of the distribution of emptiness sites in the analyzed resin were generated for each process.

### 2.3. Biological Evaluation of Materials Dedicated to Atraumatic Surgical Instruments

#### 2.3.1. Cytotoxicity and Analysis of Pro-Inflammatory Molecules

**Figure 1:**
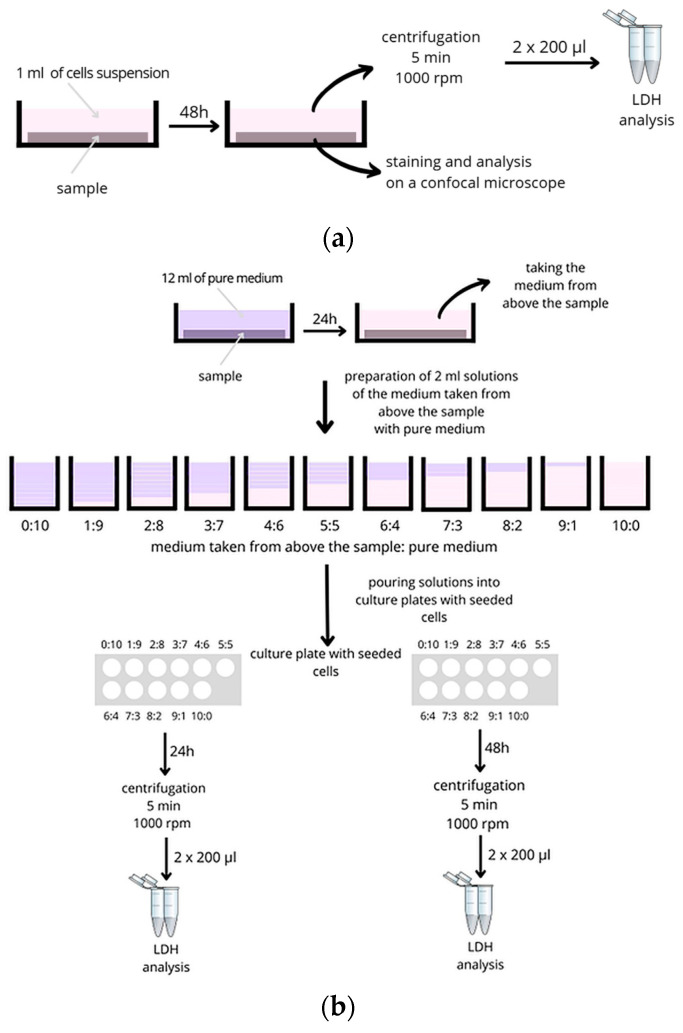
Scheme: (**a**) Direct cytotoxicity; (**b**) cytotoxicity on extracts.

Biocompatibility was determined by direct cytotoxicity tests as well as by cytotoxicity tests on extracts, according to PN-EN ISO 10993-5 standard [45]. Cytotoxicity studies were performed on Normal Human Dermal Fibroblasts (NHDF, Promocell, Heidelberg, Germany). The results were related to the biocompatibility of the reference substrate, an unmodified polystyrene (PS) considered inert in biological systems. Fibroblast Growth Medium 2 Ready-to-use (C 23010, PromoCell, Heidelberg, Germany) was used as the culture medium, enriched with Supplement Mix (PromoCell, Heidelberg, Germany) and Antibiotic Antimycotic Solution (Sigma-Aldrich, Burlington, MA, USA). The cells were seeded at a density of 105 cells/mL. Cytotoxicity was evaluated using both direct and indirect approaches, as illustrated in Figure 1.

In the direct cytotoxicity tests, cells were seeded onto specific samples, cells were also seeded on a control surface, which was polystyrene, and after 48 h, the supernatant medium above the sample was collected for evaluation of LDH (lactate dehydrogenase) enzyme and selected pro-inflammatory proteins.

The second method considered extracts. The material samples were placed in a pure medium for 24 h. In this case, the medium from above the samples was mixed with full growth medium in the ratio:

- 1 mL of medium from above the sample (10:0);
- 0.9 mL of sample extract and 0.1 mL of full growth medium (9:1);
- 0.8 mL of sample extract and 0.2 mL of full growth medium (8:2);
- 0.7 mL of sample extract and 0.3 mL of full growth medium (7:3);
- 0.6 mL of sample extract and 0.4 mL of full growth medium (6:4);
- 0.5 mL of sample extract and 0.5 mL of full growth medium (5:5);
- 0.4 mL of sample extract and 0.6 mL of full growth medium (4:6);
- 0.3 mL of sample extract and 0.7 mL of full growth medium (3:7);
- 0.2 mL of sample extract and 0.8 mL of full growth medium (2:8);
- 0.1 mL of sample extract and 0.9 mL of full growth medium (1:9);
- 1 mL full growth medium (0:10).

The prepared solutions were then used as a culture medium for fibroblasts. Cells were cultured (37 °C, 5% CO_2_) for 24 and 48 h, and next the supernatant was collected into 2 mL Eppendorf tubes, centrifuged (5 min, 1000 rpm), aliquoted for 200 μL, and stored at −80 °C for evaluation of LDH and selected pro-inflammatory proteins.

The LDH assay was performed according to the manufacturer’s protocol (CytoTox 96^®^ Non-Radioactive Cytotoxicity Assay, Promega, Madison, WI, USA) and Cytation 5 microplate reader (Agilent, Santa Clara, CA, USA).

Evaluation of selected inflammatory-related proteins (PTX3, IL-6, IL8, IL-23) in cell culture media was performed with the use of Bio-Techne Discovery Luminex assay and MagPix Limunex System (Austin, Fitchburg, MA, USA).

#### 2.3.2. Microbiology Analysis

Fluorescence in situ hybridization (FISH) is a molecular biology technique that allows visualization of microorganisms directly in situ using fluorescently labelled probes designed to bind to specific microbial ribosomes [45,46,47,48]. It is also used in diagnostics since the time-to-result is relatively short (hours) compared to standard microbiological techniques (which take days). Using confocal microscopy, we can observe the 3D structure of biofilms. The specificity of the probes used in this study was examined by hybridization with target and non-target strains, as previously evaluated [49,50]. All oligonucleotide probes used were specific to their respective target strains, as determined by 35% formamide. The biofilm was dehydrated by placing samples in 50%, 80% and 96% ethanol solutions for 5 min each. For *E. coli*, a Cyanine 3 labelled DNA probe (pB-3938) was used, resulting in an orange color, and for S. aureus, a probe (pB-349) labelled with Cyanine 5, resulting in a red color, was used. Both probes were synthesized and HPLC-purified by Biomers (Ulm, Germany). Probes were added in appropriate amounts to a freshly prepared hybridization buffer containing 32.5% formamide (Sigma-Aldrich, Burlington, MA, USA) in order to create a final concentration of 50 ng/µL. The solution was applied on the samples and they were incubated for 90 min at 46 °C and 80% humidity, protected from light. Meanwhile, a washing buffer was prepared and warmed up to 48 °C just before the incubation time ended. The samples were moved to the washing buffer and incubated for 20 min at 48 °C. Then, the samples were carefully rinsed twice with sterile Millipore water that was previously cooled to 4 °C and stored at 4 °C prior imaging. Staining was analyzed using a confocal laser scanning microscope (CLSM) equipped with LD EC Epiplan-Neofluar 50× (Carl Zeiss Microscopy Deutschland GmbH, Oberkochen, Germany) in order to detect bacterial biofilm formation. The FISH procedure, visualization and documentation of the results were performedas described previously [44,48].

### 2.4. Numerical Simulation of the Maximum Forces Carried by the Blood Vessel FE Model of Blood Vessel Compression Test

Geometry of the blood vessel [51,52] was as follows: diameter 2.7 mm, thickness 0.7 mm, and length 10 mm. The geometry of the upper insert of the surgical instrument pressed against the large blood vessel was set as follows: total length 5 mm, width 4 mm, and thickness of 2 mm. A rough contact was established between the blood vessel walls and the inserts. Perfectly rough frictional contact [53,54,55,56] assumes no sliding. No automatic closing of gaps is performed. This case corresponds to an infinite friction coefficient between the contacting bodies. Rough contact is a non-linear contact type, for which pressure is transferred, tension is not transferred, and shear is infinite. The lower insert is stationary, meaning a fixed support boundary condition is applied. No pressure is introduced inside the blood vessel. A significant force of 4N is applied to the upper insert.

**Figure 2:**
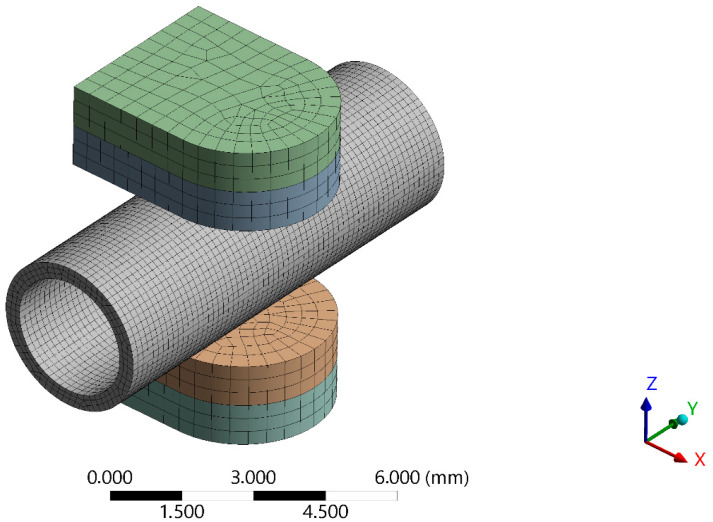
Side view of the developed computational 3D FE domain in Ansys 2024 R2 before deformation.

The developed 3D (three-dimensional) finite element (FE) mesh of the small blood vessel and insert model consisted of 76,338 nodes and 149,555 elements (Figure 2). The selected finite element mesh quality parameters are as follows: average element quality—0.9 and aspect ratio—1.47. The computed average values of the mesh quality parameters indicate that the developed finite element mesh is of very good quality. Convergence analysis was also performed for the finite element meshes generated for the loaded blood vessel model in Ansys 2024 R2. The Sparse Direct Solver [57,58] was applied to solve the defined finite element task.

Figure 2 shows the entire computational domain in which significant deformation of the blood vessel occurs; therefore, the computational mesh had to be selected properly. The mesh before and after deformation may appear distorted in the contact zone, but this is only a graphical effect; at higher magnification and from different angles, there is no distortion of the meshes. The blood vessel is most deformed, while the insert meshes remain regular, and these deformations are not significant.

**Figure 3:**
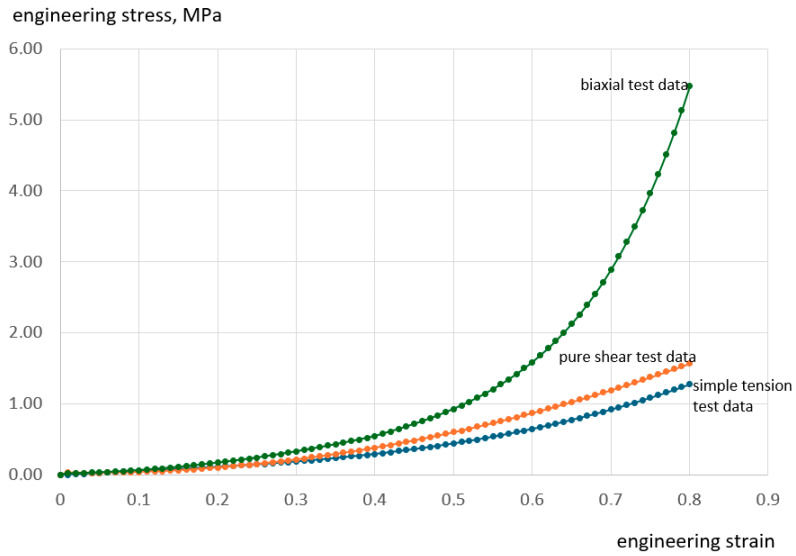
Engineering stress–strain data of the Mooney–Rivlin hyperelastic material model of the artery.

The hyperelastic material model requires the introduction of three curves from tests: uniaxial (blue), biaxial (green), and shear (orange), as presented by Dwivedi et al. [59,60]. The engineering stress–strain data were entered into the Ansys 2024 R2 program, where the Mooney–Rivlin hyperelastic material model was fitted to the artery stress–strain data (Figure 3).

The Mooney–Rivlin hyperelastic material model is one of the simplest models of the complete polynomial modes. Its SEF (strain energy density function) is given as follows [61,62,63,64]:

(1) $$W=C_{10}\left(I_{1}-3\right)+C_{01}\left(I_{2}-3\right)+\frac{1}{D_{1}}{\left(J_{el}-1\right)}^{2}$$

The model is applicable to small to moderate deformations, including up to 100% tensile strain and 30% compressive strain. Among the most used constitutive models in the literature are the Mooney–Rivlin and Ogden formulations. A key limitation is that their parameters, which lack direct physical interpretation, must be determined experimentally. Moreover, the fitting process becomes increasingly complex as the number of parameters grows [65,66].

To facilitate the selection of an appropriate hyperelastic model, the finite element software Ansys offers a curve fitting tool. This functionality streamlines the process by allowing users to compare a limited set of predefined models against experimental data, ultimately identifying the best match. To determine the relevant coefficients of the strain energy function (SEF), experimental stress–strain data must be provided. The necessary input includes results from uniaxial tension, planar shear, biaxial tension, and volumetric compression tests, which serve as the basis for deriving SEF equations expressed in terms of strain invariants or stretch ratios for hyperelastic materials. Analytical stress–strain curves were generated, and the Mooney–Rivlin model was assessed for its compatibility with the experimental results [59,60]. For the curve-fitting procedure, the most consistent data sets across all test cycles were selected [67,68]. The stress–strain data pairs from the uniaxial tensile, the planar shear, and the equibiaxial tensile tests are used to determine the shear constants *C*ij, whereas the compressibility constant, Di, is determined by the volumetric test (here assumed zero, incompressible isotropic material). For each stress–strain data pairs, the Ansys software yields the coefficients of SEF for the selected hyperplastic model.

The form of the strain-energy potential for the final nine-parameter Mooney–Rivlin hyperplastic material model applied in the present paper is:

(2) $$\begin{array}{cc} W= & C_{10}\left(I_{1}-3\right)+C_{01}\left(I_{2}-3\right)+{C_{20}\left(I_{1}-3\right)}^{2}+C_{11}\left(I_{1}-3\right)\left(I_{2}-3\right)+{C_{02}\left(I_{2}-3\right)}^{2}+{C_{30}\left(I_{1}-3\right)}^{3}\\ & \;\;\;+{C_{21}\left(I_{1}-3\right)}^{2}\left(I_{2}-3\right)+{C_{12}\left(I_{1}-3\right)\left(I_{2}-3\right)}^{2}+{C_{03}\left(I_{2}-3\right)}^{3}+\frac{1}{D_{1}}{\left(J_{el}-1\right)}^{2} \end{array}$$

where material constants are *C*_10_ = 0.19077 MPa, *C*_01_ = −0.12474 MPa, *C*_20_ = −0.25403 MPa, *C*_11_ = 0.62099 MPa, *C*_02_ = −0.25393 MPa, *C*_30_ = 0.19529 MPa, *C*_21_ = −0.36484 MPa, *C*_12_ = 0.21558 MPa, *C*_03_ = −0.033233 MPa, *D*_1_ = 0 MPa^−1^.

The initial shear modulus is given by:

(3) $$\mu =2\left(C_{10}+C_{01}\right)$$

The initial bulk modulus is given by:

(4) $$K=\frac{2}{D_{1}}$$

The elastic–plastic material model for Ti 6Al 4V for the upper and lower inserts of surgical instruments was assumed as follows: yield stress of 1045 MPa and tensile strength of 1083 MPa. The data was obtained in the present research project mentioned in the acknowledgments using standards ASTM B265-15 [69] and ASME SB 265-17 [70].

Computations were performed on a PC-class unit equipped with an Intel Core i9-13900K CPU, 128 GB of RAM, and an NVIDIA GeForce RTX 4070 Ti GPU, using ANSYS 2025 R1 (ANSYS Inc., Canonsburg, PA, USA).

### 2.5. Experimental Verification of Numerical Model

#### 2.5.1. Design of a Mechanical System to Assess the Strength of the Blood Vessel Wall

**Figure 4:**
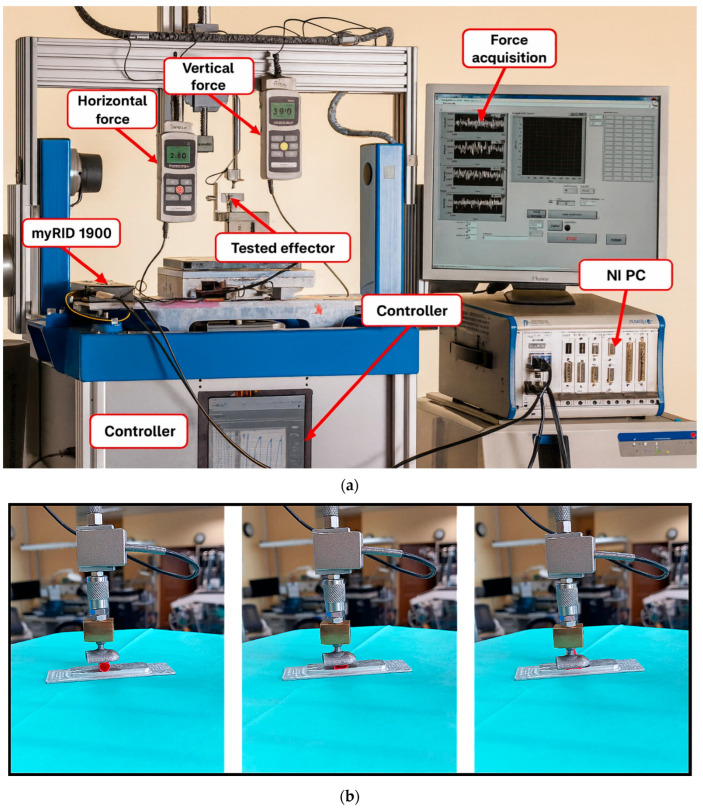
Experimental workflow of coronary artery pressure analysis: (**a**) view of the device and (**b**) images of successive experiment steps.

The traumatizing effect of the clamping forceps, as mentioned earlier, can cause damage and dissection of the vascular tissue. A dissection most often occurs due to local damage or rupture of the aortic wall on the inner side. Once the gate for the dissection is formed, two channels form in the vessel—one proper channel, where blood flows, and another—between the layers of the artery wall—where blood does not move. The abnormal channel can enlarge and compress the outlets of the aortic branches. The flow through the second normal channel is then restricted. Such a condition causes ischemia of the organs supplied by the artery. Therefore, to verify the required force order to close the flow lumen at different pressures, the previously presented numerical simulations, validated on the measurement stand, were performed. For this purpose, a 4-axis research stand equipped with three force sensors was used. The study was carried out for prepared porcine vessels (collected during slaughter for food or sanitary purposes does not require ethics committee approval due to the Act of 15 January 2015 on animal protection in science (Dz.U. 2015, poz. 266)) with a diameter in the range of Ø1.9–2.7 mm and pressures in the range p = 0–260 mmHg. The appearance of the device and images of the experiment carried out are presented in Figure 4.

#### 2.5.2. Histopathological Analysis

Initially, standard paraffin techniques were used for histological evaluation. These involve preserving the tissue structure, removing water, embedding it in wax for support, and then slicing it into thin sections of approximately 5 µm that can be stained and evaluated under a microscope. Verhoeff–van Gieson staining using kit HT25A (Sigma-Aldrich, Burlington, MA, USA) was performed to visualize and differentiate elastin fibers (dark blue/black) from collagen fibers (pink), allowing analysis of their structure and organization in histological images. Verhoeff’s hematoxylin is applied to overstain the tissue, differentiated with ferric chloride, leaving elastin and nuclei stained dark blue to black. Van Gieson’s solution is then used as a counterstain, coloring collagen red and other tissue elements yellow. Signs of tissue trauma, such as decreased arterial wall thickness, fiber compression, tissue delamination, and vessel dissection or rupture, can be observed by assessing the disruption of these fiber networks.

## 3. Results

### 3.1. Surface Analysis

#### 3.1.1. Topography Tests

The roughness of a surface affects its adhesive properties, its wear rate or its susceptibility to corrosion. These characteristics are crucial for assessing the suitability of materials for surgical instruments, as they must exhibit high corrosion resistance, the ability to be repeatedly sterilized, and reliability, among others. In the present study, three-dimensional roughness measurement according to ISO 25178-70 [71] was used to analyze the surfaces of the materials tested—BioMed 50A and BioMed 80A. This method is non-contact, faster, and more efficient than contact methods of roughness measurement; however, it is often less accurate. The surface roughness parameters that were determined were Sq, Sp, Sv, Sz, Sa. The parameter Sq is the mean square surface roughness, Sp is the maximum height of vertices, Sv is the maximum depth of depressions, the parameter Sz is defined as the maximum height from the highest point to the deepest valley, while Sa is the arithmetic mean surface roughness. Based on 3D images obtained with a digital microscope, the roughness of the lateral surfaces of the samples was analyzed in ConfoMap® Surface Imaging and Analysis Software for ZEISS microscopes V10 (Carl Zeiss, Jena, Germany) according to ISO 25178-1 [71]. The roughness parameters analyzed were Sq, Sp, Sv, Sz, and Sa. The results are presented in Table 2.

Lower roughness Sq is shown by the BioMed 80A material (3.20 µm). For the parameter Sv, which defines the depth of the lowest valley, BioMed 80A shows a higher value (10.82 µm), confirming a higher surface irregularity compared to BioMed 50A (8.64 µm). The Sz and Sa parameters show small differences between Formlabs 50A and Formlabs 80A. As for the Sp parameter, Formlabs 50A shows a higher value.

#### 3.1.2. Contact Angle

Results of contact angle measurements are presented in Table 3, Table 4, Table 5 and Table 6. The obtained Surface Free Energy results are presented in Table 7, Table 8, Table 9 and Table 10.

The surface characteristics of both analyzed materials—Biomed Elastic 50A and Biomed Flex 80A—prior to sterilization are hydrophobic, as all measured contact angles exceed 90°.

Analyzing the contact angle values for Biomed Elastic 50A (Table 3), it can be observed that ethylene oxide sterilization does not affect surface characteristics—after three cycles, the surface retains its hydrophobic nature. In contrast, autoclave sterilization changes the surface character from hydrophobic to hydrophilic (Table 4). The average contact angle value for the material before sterilization is 102.0°, while after three sterilization cycles, it decreases to 67.6°. Differences in contact angle values for water and PBS are not significant for either material, ranging from 3.9% to 7%.

For Biomed Flex 80A, samples sterilized with ethylene oxide exhibited a hydrophilic surface character, while the contact angle for samples sterilized in an autoclave showed values below 90°, also suggesting the material’s hydrophilicity. The contact angle values for both sterilization methods did not differ significantly (differences for distilled water ranged from 2% to 6%). For autoclaved samples, slight differences in contact angle were observed, with the contact angle decreasing as the number of sterilization cycles increased. In samples sterilized with ethylene oxide, it was not possible to clearly determine the effect of the number of sterilization cycles on surface wettability, as the contact angles changed only slightly.

For ethylene oxide-sterilized samples, the surface energy parameter (SEP) increased with the number of sterilization cycles. In contrast, for autoclaved samples, no clear correlation between SEP and the number of sterilization cycles was observed, as SEP values remained similar across cycles. A high value of the dispersive component (γSD) of the SEP for each analyzed sample may indicate a hydrophobic surface character of the materials.

The results of the conducted studies indicate that sterilization, whether with ethylene oxide or in an autoclave, has a minimal effect on the surface characteristics of Biomed Flex 80A. However, for Biomed Elastic 50A, autoclave sterilization notably alters the surface properties, rendering them hydrophilic.

### 3.2. Non-Destructive Analysis of the Risk of Creating Voids in the Material in the Emergence of Acoustic Tools

**Figure 5:**
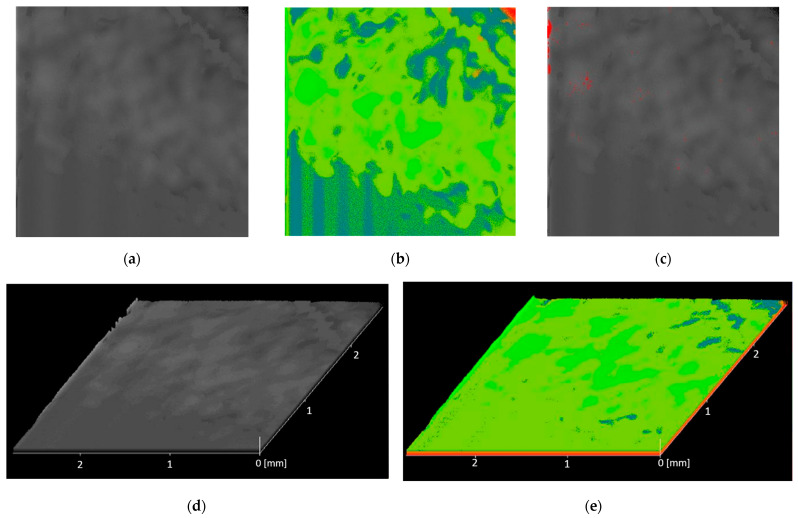
Results of 50A SAM analysis: (**a**) 2D topography—gray, (**b**) 2D topography—color, (**c**) 2D delamination, (**d**) 3D topography—gray, (**e**) 3D topography—color.

**Figure 6:**
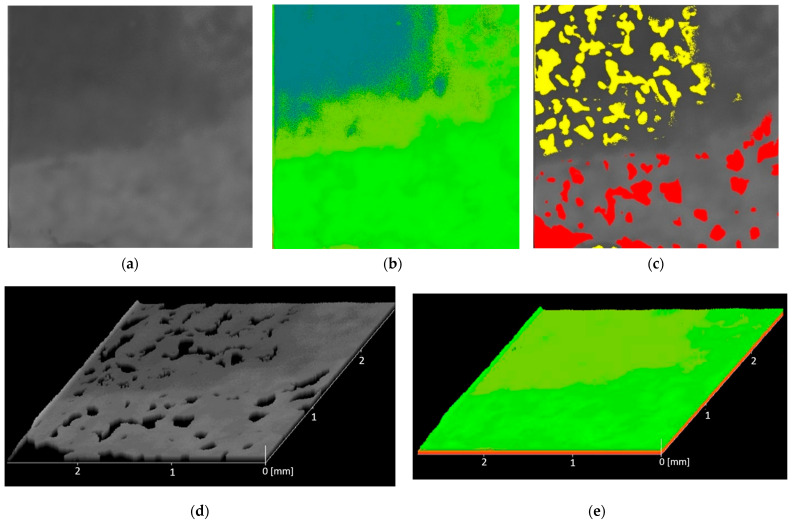
Results of 80A SAM analysis: (**a**) 2D topography—gray, (**b**) 2D topography—color, (**c**) 2D delamination, (**d**) 3D topography—gray, (**e**) 3D topography—color.

Results of scanning acoustic microscopy analysis, including 2D, 3D, and delamination, are presented below (Figure 5 and Figure 6).

### 3.3. Biological Evaluation of Materials Dedicated to Atraumatic Surgical Instruments

#### 3.3.1. Cytotoxicity Lactate Dehydrogenase Levels

**Figure 7:**
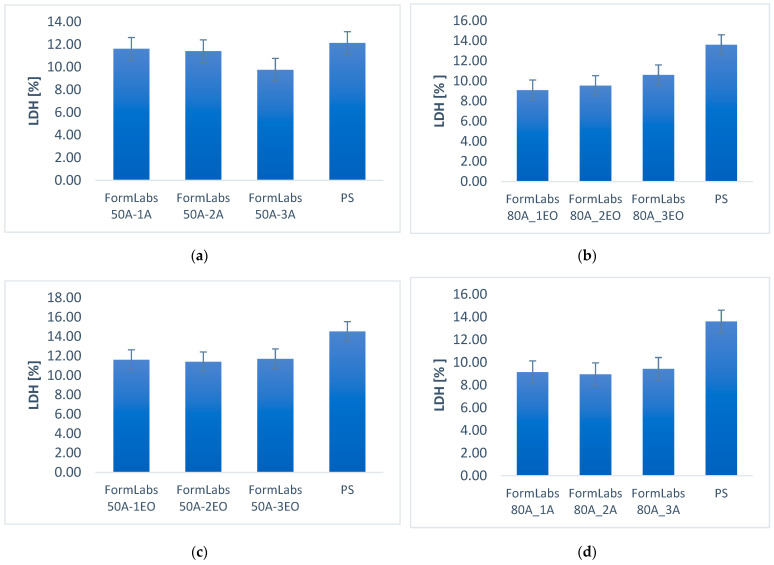
LDH level in cell culture medium after cytotoxicity in direct contact: (**a**) FormLabs 50A autoclave; (**b**) FormLabs 80A autoclave; (**c**) FormLabs 50A ethylene oxide; (**d**) FormLabs 80A ethylene oxide.

The results of direct lactate dehydrogenase testing are shown in Figure 7. The results show the levels of the released isoenzyme from cells in direct contact with the test material surface. Tests were conducted on two FormLabs 50A and 80A materials using two sterilization methods: steam and ethylene oxide. Three sterilization cycles were performed for each sterilization method.

All LDH levels are below the reference for PS, which means that the material after each type of sterilization does not adversely interact with cells.

**Figure 8:**
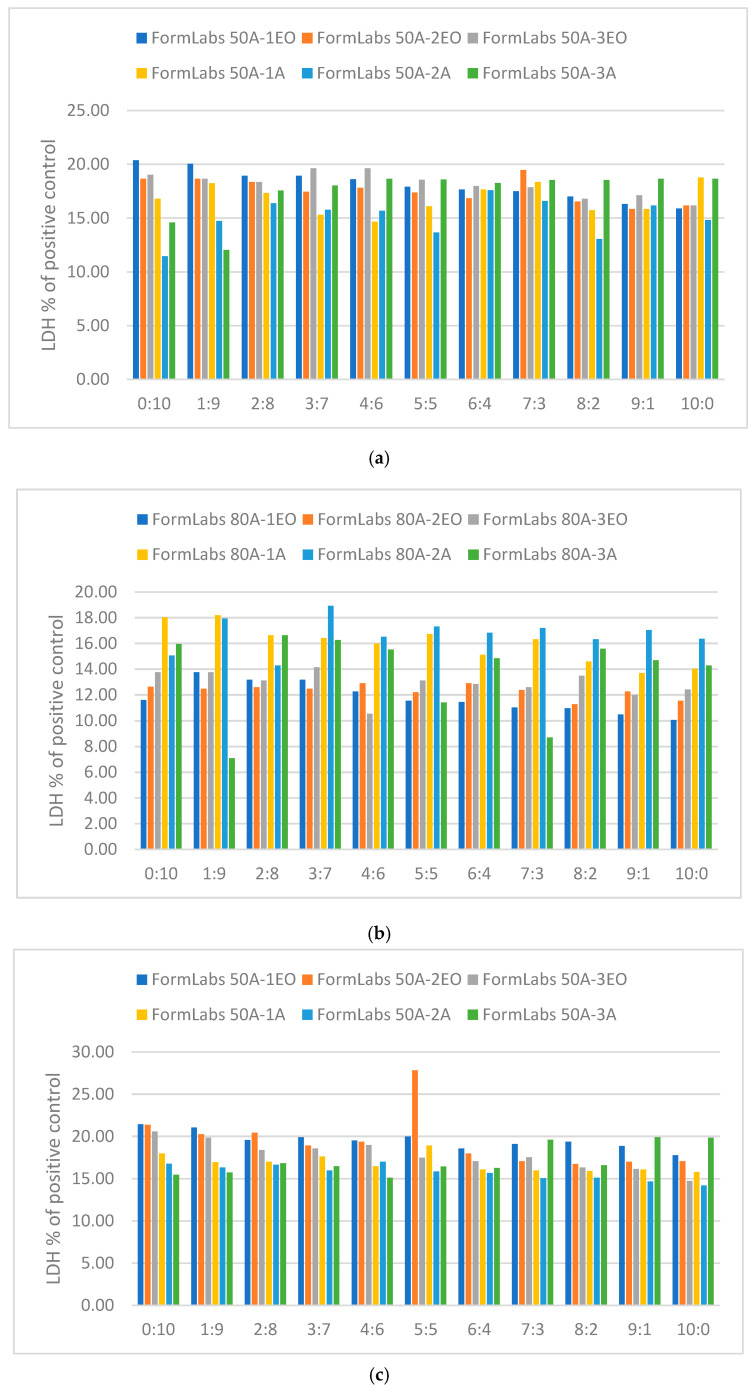

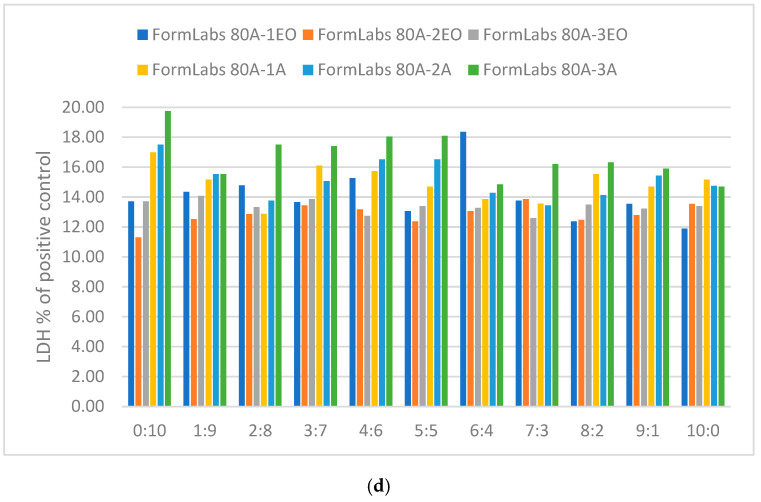
LDH level in cell culture medium after cytotoxicity on extracts: (**a**) FormLabs 50A 24 h; (**b**) FormLabs 80A 24 h; (**c**) FormLabs 50A 48 h; (**d**) FormLabs 80A 48h.

The results of the lactate dehydrogenase extract testing are shown in Figure 8.

All LDH levels are within acceptable limits compared with the positive control, indicating that the extracts from specimens after each type of sterilization do not adversely interact with cells. A rapid increase in LDH value is observed for dilution 5:5 for FormLabs 50A-EO; however, it is still within the acceptance level.

#### 3.3.2. Analysis of Pro-Inflammatory Molecules

**Figure 9:**
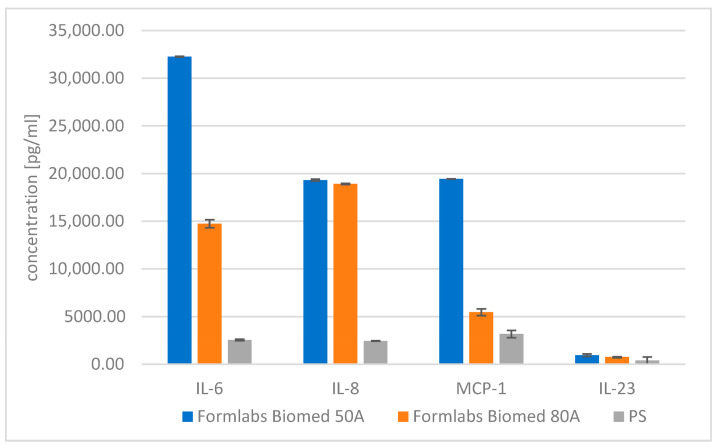
Concentration of selected cytokines of tested materials—direct method.

**Figure 10:**
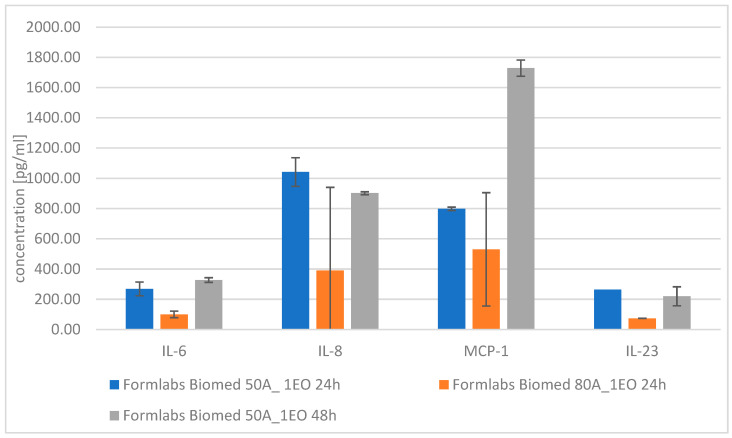
Concentration of selected cytokines of tested materials—extract method.

Results of direct (Figure 9 and Table 11) and indirect exposure (extract method, Figure 10 and Table 12) of the tested materials on the production of pro-inflammatory proteins by fibroblasts are presented below.

Of the thirteen cytokines, seven (TNF-α, IL-10, IL-1β, GM-CSF, CD-62p, IFN-γ, IFN-α) were below the manufacturer’s detection limit.

**Figure 11:**
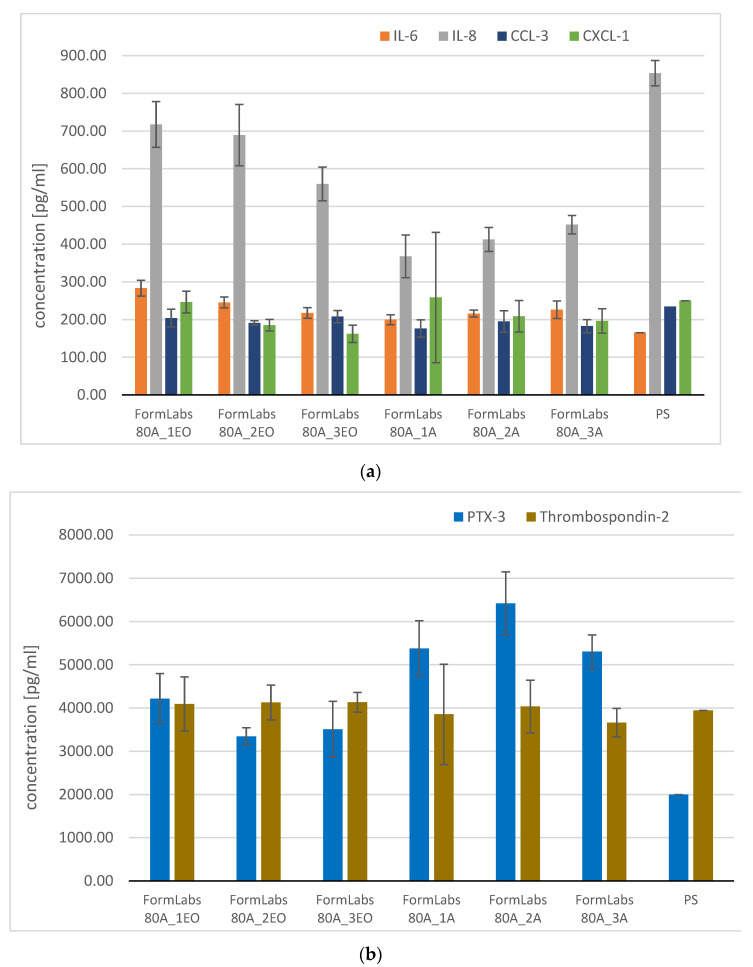

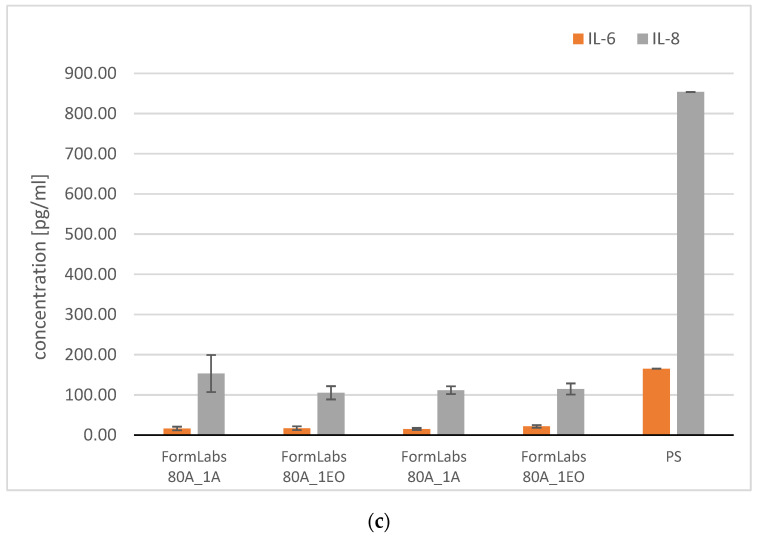
Concentration of selected cytokines for tested materials: (**a**) IL-6, IL-8, CCL-3, CXCL-1, (**b**) PTX-3 and Thrombospondin-2, (**c**) extract method: IL-6 and IL-8 (dilution:10:0).

The highest observed differences were recorded for IL-8, in the case of EO sterilization (Figure 11a). Its concentration decreased with increasing cycles (from 717 pg/mL for 1 cycle to 559 pg/mL for 3 cycles). However, it was still higher than in the autoclaving case, where the concentration increased (from 367 pg/mL for 1 cycle to 451 pg/mL for 3 cycles). Similar observations were made for the IL-6 cytokine (Figure 11a). Concentration of CCL-3 (Figure 11a) was around 200 pg/mL for all tested samples; the differences were within the standard deviation (~ 20 pg/mL). For CXCL-1, both tested sterilization methods showed lower concentrations with each cycle (Figure 11a).

In the case of Thrombospondin-2 (Figure 11b), all noted concentrations were around 4100 pg/mL and did not differ significantly from control. The PTX-3 concentration (Figure 11b) was significantly higher after autoclave sterilization (>5300 pg/mL) than autoclave (~4000 pg/mL), whereas for the control it was around 2000 pg/mL.

#### 3.3.3. Microbiological Analysis

**Figure 12:**
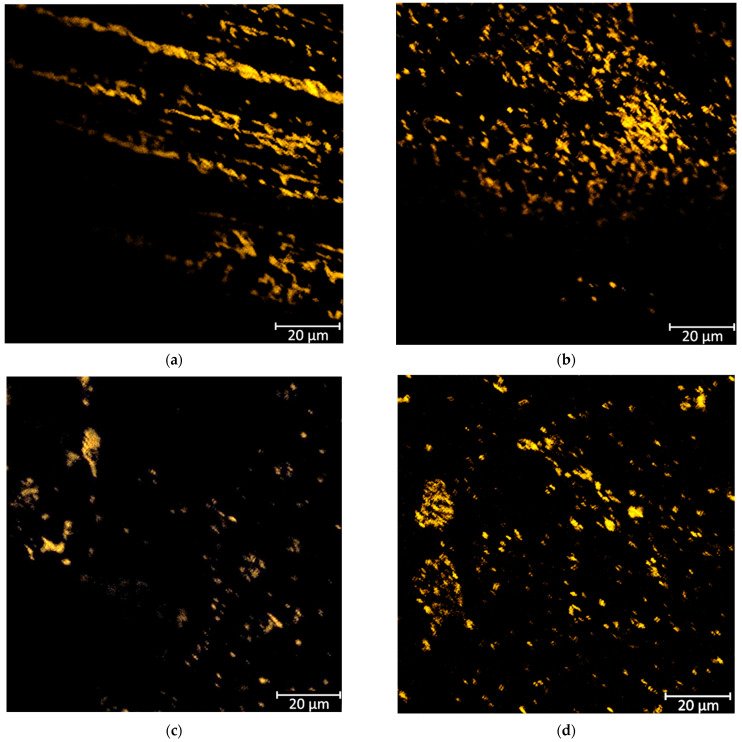
*E. coli* FISH staining of tested materials: (**a**) 80A_1A, (**b**) 80A_1EO, (**c**) 50A_1A, (**d**) 50A_1EO.

**Figure 13:**
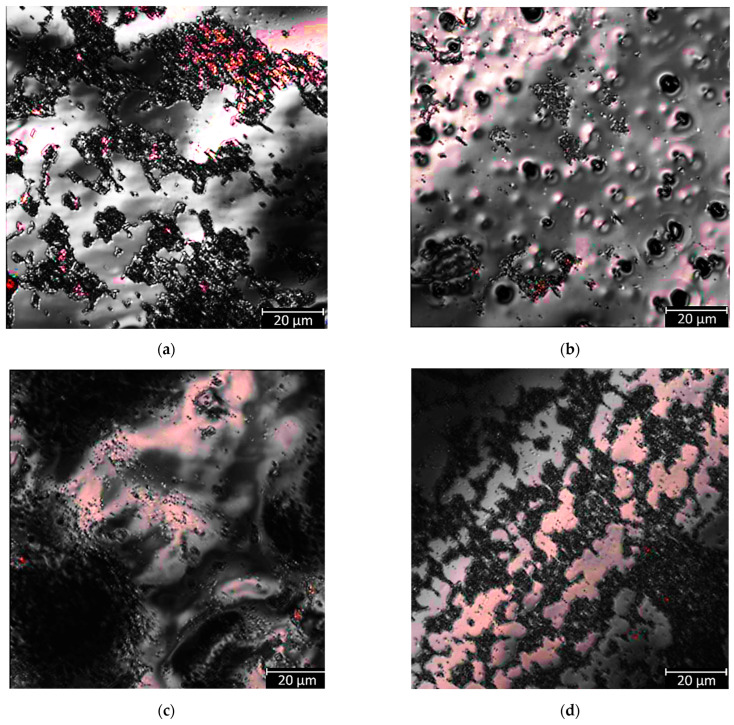
S. aureus FISH staining of tested materials: (**a**) 80A_1A, (**b**) 80A_1EO, (**c**) 50A_1A, (**d**) 50A_1EO.

Results of FISH imaging are presented in Figure 12 and Figure 13. On the autoclaved surface of the 80A material, more S. aureus bacterial cells are observed; the bacteria form large clusters, and there are large uncolonized spaces between them. On the contrary, observations in the case of the 50A are the opposite—there are more S. aureus cells on the surface previously sterilized with ethylene oxide; moreover, the bacteria are more evenly distributed in smaller clusters, with neither larger clusters nor spaces free from colonization. For 80A material, there are no noticeable differences in the number of E. coli cells across sterilization methods; moreover, there are noticeable uncolonized areas. In the 50A case, there are slightly more E. coli cells on the previously sterilized surface with ethylene oxide; uncolonized spaces are also noticeable.

Escherichia coli cells are about 0.5 × 2 μm. In this case, they were marked with probes containing Cyanine 3 dye, which results in an orange fluorescence signal. For 80A, no noticeable differences between the number of *E. coli* bacteria and the sterilization method were observed. However, uncolonized spaces (Figure 12b) for material 50A were noticeable. There are slightly more *E. coli* bacteria on the surface previously sterilized with ethylene oxide, with noticeable uncolonized spaces.

Staphylococcus aureus cells are about 1 micrometer and form clusters. For this study, Cyanine 5 marked probes were used, which resulted in a red fluorescence signal. In the case of material 80A, there are cells of S. aureus bacteria on the autoclave-sterilized surface and the bacteria form large clusters. There are large uncolonized spaces between the clusters (Figure 13b) for 50A material; more bacterial cells are present on the previously sterilized surface.

### 3.4. Numerical Simulation of the Maximum Forces Carried by the Blood Vessel

During simulation development, mesh quality parameters were calculated; however, their distributions were uniform, and no zones requiring further improvement were identified. Therefore, the distributions of mesh quality parameters of the computational 3D FE grid are not shown in this paper (the average mesh quality parameters were presented in Section 2.4).

**Figure 14:**
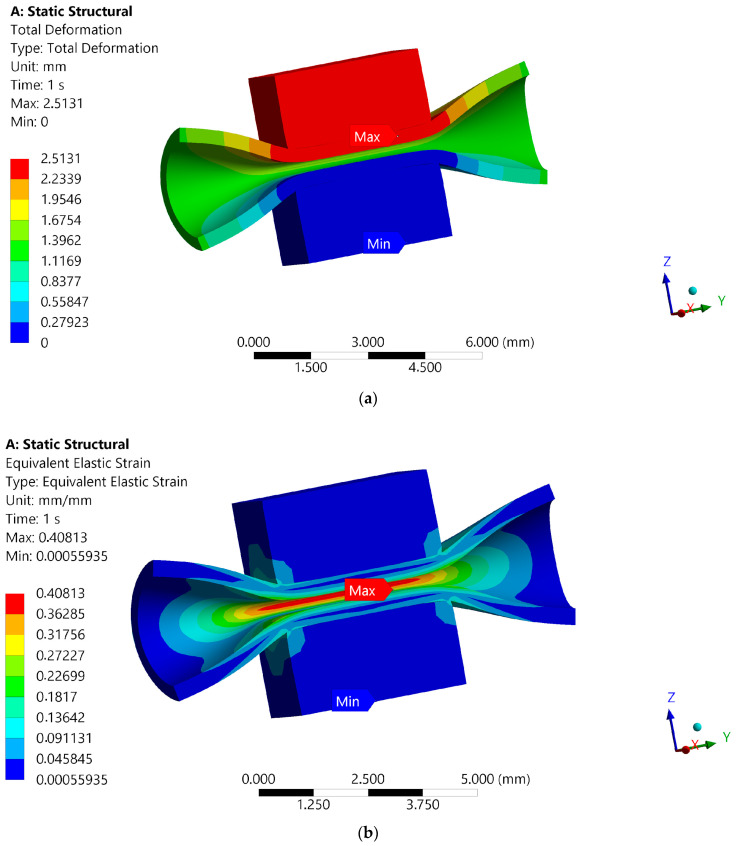
Distributions of (**a**) total deformation, in mm, and (**b**) an equivalent elastic strain, without unit.

**Figure 15:**
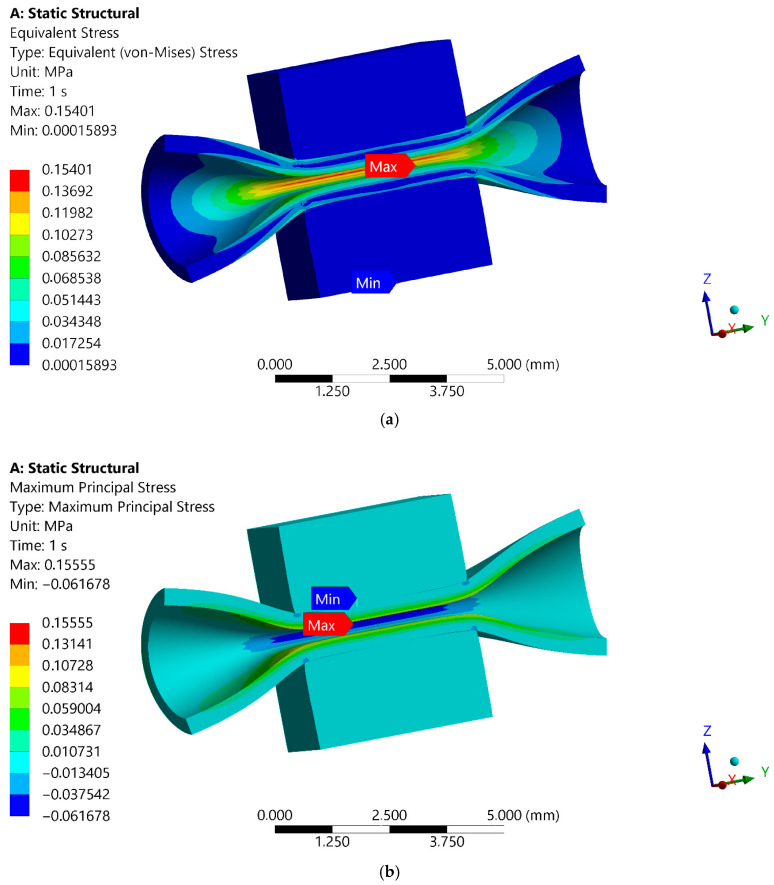
Distributions of (**a**) equivalent von Mises stress, in Mpa, and (**b**) principal stress, in MPa.

**Figure 16:**
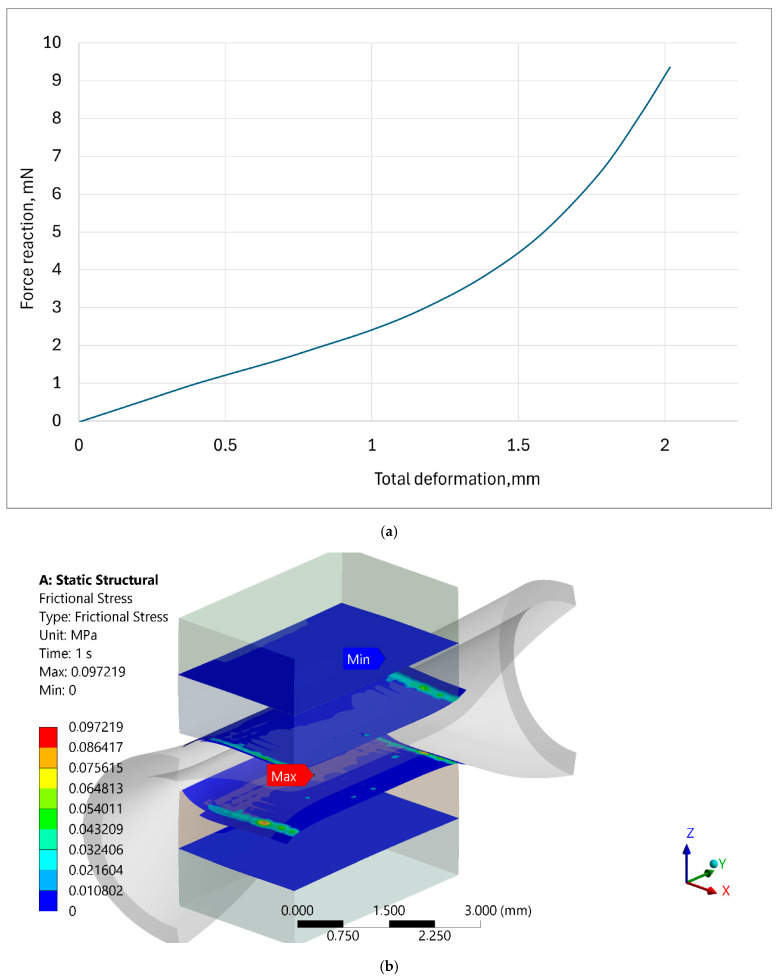
(**a**) Curve representing force reaction versus total deformation in the blood vessel–insert contact zone, in N and in mm. (**b**) Distribution of frictional stress in the blood vessel–insert contact zone, in MPa.

The results of 3D FE simulations are presented as distributions of total deformation (mm) and equivalent elastic strain (no units; Figure 14), as well as distributions of equivalent von Mises stress (MPa) and principal stress (MPa; Figure 15). Due to the selected rough contact between the artery and the inserts, frictional stress (MPa) and contact force (N) are also computed and reported numerically (Figure 16).

The results from the numerical model of the blood vessel compression test can be discussed in terms of the critical strain and stress values shown in the material models of the blood vessel and the insert, as there are no similar solutions in the literature. The distributions of total deformation and equivalent elastic strain in the contact zone between the blood vessel and the inserts are shown in Figure 14. The upper insert transfers displacements, but the compressed artery deforms the most, with a maximum strain of 0.4. This maximum strain value is not critical for the hyperelastic blood vessel. The lower insert of the surgical instrument does not deform. In terms of calculated stresses, the highest values are also observed in the blood vessel wall (Figure 15). A specific stress value computed in the upper insert results from friction at the blood vessel–insert contact zone and occurs symmetrically on both sides of the model. The maximum value of equivalent von Mises stress is 0.154 MPa in the blood vessel. The maximum value of principal stress in the 3D FE model is 0.155 MPa. These maximum stress values are not critical for the hyperelastic material of the blood vessel. The maximum value of reaction force in the blood vessel–insert contact zone is 0.09 N (Figure 16). The maximum value of frictional stress in the blood vessel–insert contact zone in the 3D FE model is 0.097MPa (Figure 16). The computed values of both these contact parameters (frictional stress and reaction force) are low.

### 3.5. Experimental Verification of Numerical Model

#### 3.5.1. Effect of Applied Force on Coronary Artery Pressure

**Figure 17:**
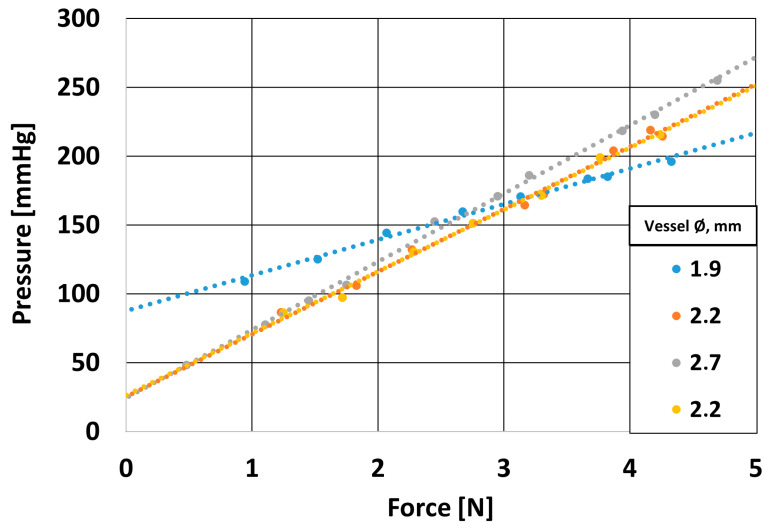
Effect of applied force on coronary artery pressure.

The results presented in the graph are highly relevant to vascular clamps used during surgical procedures (Figure 17). During surgical procedures, it is often necessary to temporarily occlude blood vessels to prevent blood loss and enable the surgeon to perform precise maneuvers. The selection of an appropriate clamping force is crucial—it must be sufficient to completely close the vessel lumen and stop blood flow but controlled enough to avoid permanent damage to the vessel wall, which could lead to postoperative complications. The graph shows that the force required to achieve a given pressure increases with the vessel diameter. To completely occlude the flow in an artery with a diameter of approximately 2.2 mm, a clamping force of 2.5–3 N is required; for larger vessels, such as those with a diameter of 2.7 mm, this force increases to around 4 N. In the case of smaller vessels, such as those 1.9 mm in diameter, blood flow can be effectively blocked with a force of about 2 N. The study demonstrates that the clamping force largely depends on the vessel’s internal pressure and diameter. For example, in peripheral arteries, the required clamping force typically ranges from 2 to 5 N, whereas in veins—where blood pressure is much lower (5–15 mmHg)—occlusion can often be achieved with forces below 2 N. This highlights the importance of designing surgical clamps with materials and structures that can compensate for mechanical stress while ensuring atraumatic occlusion. Since higher intravascular pressure and larger vessel diameters require greater clamping forces, the choice of clamp must account for these factors to prevent excessive compression that could lead to tissue damage. Advanced clamp designs should incorporate adaptive mechanisms or flexible materials that evenly distribute pressure, minimizing the risk of vessel trauma. These findings are particularly relevant for vascular, transplant, and cardiac surgery, where precise occlusion with minimal tissue injury is critical for successful surgical outcomes.

#### 3.5.2. Histopathological Analysis

**Figure 18:**
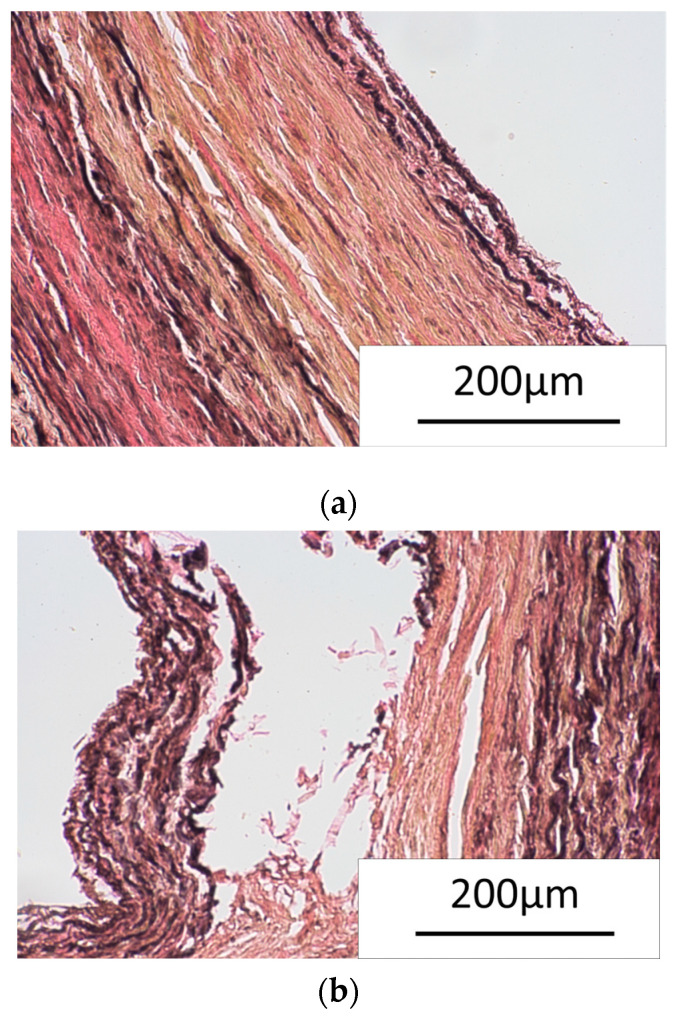
Histological analysis of the tissue (**a**) native tissue, not stressed (**b**) tissue after pressure sampling showing stratification of the blood vessel wall.

Histological examination revealed delamination of the blood vessel wall following a tool pressure test. The results presented in Figure 18a illustrate a native vessel versus a tissue stressed beyond its maximum force (Figure 18b), demonstrating damage as a vessel wall dissection. This traumatization resulted in reduced arterial wall thickness and fiber compression. Varying degrees of tissue delamination were observed, with some areas showing minimal separation and others exhibiting extensive splits between the vessel wall layers. The most affected areas exhibited signs of vessel dissection, characterized by a more extensive separation along the wall, and even ruptures, indicating a complete tear, were apparent. These findings highlight significant structural alterations caused by the mechanical stress of the tool pressure test. The comparison in Figure 18 clearly shows the impact of exceeding the vessel’s mechanical limits. The observed morphological changes, including wall thinning and compression of its fibrous components, underscore the direct physical consequences of the applied pressure. The varying levels of delamination further illustrate the progressive damage to the vessel’s integrity. The histological analysis confirmed that Verhoeff–van Gieson staining is a sufficient method for revealing tissues affected by trauma. Moreover, it was unequivocally demonstrated that excessive force during the tool pressure test induces substantial damage to the blood vessel wall. This damage includes reduced thickness, fiber compression, and diverse tissue delamination, with dissection or rupture occurring in critical cases.

## 4. Discussion

Based on simulations and experimental verification, the results showed a high risk of blood vessel wall stratification when the maximum force applied to the tool is exceeded. Surgical experience plays a significant role in closing a blood vessel so that there is no flow, but also no excess force on the vessel wall. However, some instrument features can make this task easier and more controllable. To prevent vascular defects, a metamaterial structure was proposed in both theoretical and experimental simulations. The metamaterial designed for atraumatic inserts should act as a buffer on the blood vessel to compensate for compressive stresses.

Forces acting on the blood vessel wall may reduce wall thickness, compress fibers, and cause tissue delamination. The latter may lead to blood vessel dissection or even rupture. The main problem during cardiovascular and thoracic surgeries is tissue traumatization, leading to serious adverse events, interrupting blood flow and life-threatening hemorrhage. Pressure-matching soft materials and sensor-based solutions are lacking to reduce tissue trauma and provide the surgeon with feedback data. As the commonly used cardiovascular and thoracic clamping and grasping instruments are not real-time pressure-controlled devices, almost all vascular procedures bear risks of excessive, uneven clamping force. Atraumatic materials currently lack overload protection, especially when reversible deformation exceeds the allowable limits of surgical forces. Therefore, the tool design proposes the use of metamaterials, that is, materials whose properties depend on their structure at scales larger than the molecular scale, not just on their molecular structure. The term, in particular, is used to describe materials with properties not found in naturally occurring materials. Metamaterials are often constructed from unit cells (UCs), arranged in three dimensions to form a variety of geometric profiles. The first step is the design of each UC and the definition of geometric parameters. While it enables the creation of highly variable structures, it adds complexity to the design process. Specific scenarios (isotropic or orthotropic configurations) are advised to simplify the workflow by following several reductions.

The stiffness of a material (or a structure) is still a critical factor in product development. The growing fascination with adaptable materials with variable mechanical behavior continues to shape modern design philosophies. Advancements in materials science enabled modification of such properties through external influences.

The biocompatibility of Formlabs Biomed materials in biomedical applications is influenced by several critical factors, including material composition, post-processing techniques, and their interactions with biological systems. Understanding these elements is essential for optimizing their use in medical technologies.

Material Composition

- The chemical makeup of Formlabs BioMed resins significantly affects their biocompatibility. Variations in surface properties and degradation behavior can lead to different cellular responses, such as adhesion and proliferation [72].
- The presence of non-polymerized monomers can induce cytotoxicity, necessitating careful selection of materials and processing methods [73,74].
- Post-Processing Techniques
- Effective post-processing, such as soaking in solvents like isopropanol and methanol, can significantly enhance biocompatibility by removing toxic residues [73,74].
- Additional UV exposure during post-processing can improve the polymerization degree, further reducing cytotoxic effects [72].
- Biological Interactions
- The interaction between biomaterials and biological systems is complex, influenced by genetic and environmental factors, which can lead to variability in biocompatibility outcomes [75].
- Comprehensive assessment methodologies are crucial for understanding these interactions and ensuring safety in clinical applications.

While these factors are pivotal, it is also important to recognize that biocompatibility is not solely determined by material properties; patient-specific factors and the context of use can significantly influence outcomes.

The present work focused on evaluating the cytotoxicity of the materials using direct and indirect tests, enabling assessment of their effects on cell membrane integrity and lactate dehydrogenase secretion. The results show that in both the 24 h and 48 h tests, the tested materials do not exhibit a significant cytotoxic effect, a favorable result for their potential use as biomaterials. A slight increase in LDH levels in the case of FormLabs BioMed 50A may indicate specific interactions between this material and cells, but further studies are required to better understand these phenomena. Similar observations were made by Ramaraju et al. [76] when evaluating the impact of different temperatures of EO sterilization on elastomers dedicated for biomedical applications—they observed lower cell count number, but no differences in live-to-dead cell ratio.

As part of the present work, preliminary studies of the surface interactions of selected materials with different bacterial strains were conducted. Differences in the colonization of S. aureus and *E. coli* were observed depending on the material type and sterilization method, with implications for the design of biomedical materials with antimicrobial properties. These results suggest that appropriate surface modification can significantly affect bacterial interactions with the material, which is crucial for developing safe surgical instruments and implants. There are no studies available that include FISH analysis of similar materials published in the last 15 years, which is an undoubted advantage of this paper.

Preliminary histopathological results from mechanical tests show vessel wall delamination, which is crucial for understanding the mechanisms of material–tissue interaction and the material’s potential biocompatibility.

The strain and stress values computed in the 3D FE model of compressed blood vessels by the surgical instrument inserts are not critical for either the insert or the blood vessel under the tested conditions.

## 5. Conclusions

The present study demonstrates that clamping force depends primarily on the vessel’s internal pressure and diameter. Localized wall delamination of the blood vessel was observed under excessive pressure. These findings underline the importance of mechanical force control and material elasticity in minimizing vascular trauma during surgical procedures. Consequently, the study evaluated the physicochemical and biological properties of two elastomeric materials—BioMed Flex 50A and BioMed Flex 80A—intended for use in non-traumatic surgical instruments. The results demonstrated that cytotoxicity was not affected by the number of sterilization cycles, confirming the materials’ biocompatibility under the tested conditions. However, the sterilization method influenced bacterial colonization, underscoring that surface characteristics and sterilization method play crucial roles in microbial interactions. Notably, the 80A material exhibited greater stability and lower susceptibility to sterilization-induced changes compared to 50A, indicating its superior potential for reusable surgical applications.

Overall, this study provides valuable insights into the mechanical behavior, cytocompatibility and bacterial response of the tested materials, supporting their further development as biocompatible, pressure-adaptive metamaterial components for atraumatic surgical instruments.

## Figures and Tables

**Table 1:** List of analyzed materials depending on material type, sterilization type, and number of sterilization cycles.

Sample Type	Sample Condition
Formlabs Biomed Elastic 50A	Before sterilization—Formlabs 50A
	After 1 cycle of ethylene oxide (EO) sterilization—Formlabs 50A_1EO
	After 2 cycles of ethylene oxide (EO) sterilization—Formlabs 50A_2EO
	After 3 cycles of ethylene oxide (EO) sterilization—Formlabs 50A_3EO
	After 1 cycle of autoclave (A) sterilization—Formlabs 50A_1A
	After 2 cycles of autoclave (A) sterilization—Formlabs 50A_2A
	After 3 cycles of autoclave (A) sterilization—Formlabs 50A_3A
Formlabs Biomed Flex 80A	Before sterilization—Formlabs 80A
	After 1 cycle of ethylene oxide (EO) sterilization—Formlabs 80A_1EO
	After 2 cycles of ethylene oxide (EO) sterilization—Formlabs 80A_2EO
	After 3 cycles of ethylene oxide (EO) sterilization—Formlabs 80A_3EO
	After 1 cycle of autoclave (A) sterilization—Formlabs 80A_1A
	After 2 cycles of autoclave (A) sterilization—Formlabs 80A_2A
	After 3 cycles of autoclave (A) sterilization—Formlabs 80A_3A

**Table 2:** Results of roughness measurements.

Parameter [µm]	BioMed 50A	BioMed 80A
Sq	3.52 ± 0.45	3.20 ± 0.65
Sp	13.92 ± 2.87	11.62 ± 2.18
Sv	8.64 ± 1.92	10.82 ± 1.77
Sz	22.54 ± 2.66	22.44 ± 3.14
Sa	2.71 ± 0.30	2.56 ± 0.55

**Table 3:** Contact angle values for samples 50A after ethylene oxide sterilization.

One Cycle of Sterilization			Two Cycles of Sterilization			Three Cycles of Sterilization		
			θ [°]					
Distilled water	PBS	Diiodomethane	Distilled water	PBS	Diiodomethane	Distilled water	PBS	Diiodomethane
104.6 ± 7.7	97.7 ± 9.8	50.3 ± 4.1	107.1 ± 4.6	102.5 ± 8.8	52.8 ± 3.8	102.5 ± 7.8	98.5 ± 7.8	51.4 ± 7.6

**Table 4:** Contact angle values for samples 50A after autoclaving.

One Cycle of Sterilization			Two Cycles of Sterilization			Three Cycles of Sterilization		
			θ [°]					
Distilled water	PBS	Diiodomethane	Distilled water	PBS	Diiodomethane	Distilled water	PBS	Diiodomethane
87.6 ± 3.1	90.2 ± 5.6	48.9 ± 4.3	84.9 ± 4.6	84.2 ± 6.4	47.6 ± 5.1	73.0 ± 6.4	67.6 ± 6.4	47.4 ± 6.2

**Table 5:** Contact angle values for samples 80A after ethylene oxide sterilization.

One Cycle of Sterilization			Two Cycles of Sterilization			Three Cycles of Sterilization		
			θ [°]					
Distilled water	PBS	Diiodomethane	Distilled water	PBS	Diiodomethane	Distilled water	PBS	Diiodomethane
95.3 ± 7.3	90.2 ± 4.9	51.0 ± 7.3	91.1 ± 4.2	85.3 ± 5.4	52.5 ± 4.9	90.1 ± 2.9	85.3 ± 1.6	51.2 ± 1.7

**Table 6:** Contact angle values for samples 80A after autoclaving.

One Cycle of Sterilization			Two Cycles of Sterilization			Three Cycles of Sterilization		
			θ [°]					
Distilled water	PBS	Diiodomethane	Distilled water	PBS	Diiodomethane	Distilled water	PBS	Diiodomethane
87.4 ± 5.0	84.7 ± 3.5	47.6 ± 5.2	88.7 ± 5.3	84.1 ± 4.0	48.0 ± 5.5	84.3 ± 4.4	81.5 ± 6.3	47.1 ± 4.1

**Table 7:** Surface free energy (SFE) for samples 50A after ethylene oxide sterilization.

One Cycle of Sterilization			Two Cycles of Sterilization			Three Cycles of Sterilization		
SFE According to the Owens-Wendt Model [mJ/m^2^]								
γ_S	γ_S^d	γ_S^p	γ_S	γ_S^d	γ_S^p	γ_S	γ_S^d	γ_S^p
34.1	34.1	0.0	32.7	32.7	0.0	35.3	33.6	1.7

**Table 8:** Surface free energy (SFE) for samples 50A after autoclaving.

One Cycle of Sterilization			Two Cycles of Sterilization			Three Cycles of Sterilization		
SFE According to the Owens-Wendt Model [mJ/m^2^]								
γ_S	γ_S^d	γ_S^p	γ_S	γ_S^d	γ_S^p	γ_S	γ_S^d	γ_S^p
37.0	34.9	2.1	37.1	35.4	1.8	35.7	42.9	7.2

**Table 9:** Surface free energy (SFE) for samples 80A after ethylene oxide sterilization.

One Cycle of Sterilization			Two Cycles of Sterilization			Three Cycles of Sterilization		
SFE According to the Owens-Wendt Model [mJ/m^2^]								
γ_S	γ_S^d	γ_S^p	γ_S	γ_S^d	γ_S^p	γ_S	γ_S^d	γ_S^p
34.0	33.1	0.9	35.4	33.4	2.0	36.3	32.8	3.5

**Table 10:** Surface free energy (SFE) for samples 80A after autoclaving.

One Cycle of Sterilization			Two Cycles of Sterilization			Three Cycles of Sterilization		
SFE According to the Owens-Wendt Model [mJ/m^2^]								
γ_S	γ_S^d	γ_S^p	γ_S	γ_S^d	γ_S^p	γ_S	γ_S^d	γ_S^p
35.8	33.4	2.4	35.5	33.5	2.0	36.3	32.8	3.5

**Table 11:** Concentration of selected cytokines for the tested materials.

		IL-6	IL-8	MCP-1	IL-23
Direct cytotoxicity	Formlabs Biomed 50A	32,257.14 ± 30.18	19,317.75 ± 109.95	19,434.29 ± 5.34	947.15 ± 138.53
	Formlabs Biomed 80A	14,747.70 ± 413.98	18,902.47 ± 60.54	5464.35 ± 357.03	747.86 ± 48.31
	PS	2542.78 ± 78.44	2439.99 ± 32.09	3172.09 ± 3712.09	383.69 ± 383.69

**Table 12:** Concentration of selected cytokines for tested materials—extract method.

		IL-6	IL-33	IL-8	MCP-1	IL-23
Cytotoxicity on extracts (dilution—10:0)	Formlabs Biomed 50A_ 1EO 24 h	268.70 ± 45.63	53.82 ± 12.18	1041.84 ± 94.64	798.73 ± 11.31	264.02 ± NN
	Formlabs Biomed 80A_1EO 24 h	100.14 ± 21.69	33.75 ± 0.00	389.89 ± 550.96	530.19 ± 374.90	73.84 ± NN
	Formlabs Biomed 50A_1EO 48 h	327.37 ± 15.38	below detection level	901.75 ± 9.40	1729.21 ± 53.60	219.73 ± 62.65
	Formlabs Biomed 80A_1EO 48 h	375.43 ± 15.38	<13.90	1085.99 ± 63.82	1750.87 ± 70.67	219.73 ± 62.65
	Formlabs Biomed 50A_1A 24 h	175.09 ± 6.51	below detection level	456.56 ± 9.14	762.19 ± 1.95	124.64 ± 71.83
	Formlabs Biomed 80A_1A 24 h	169.89 ± 4.91	<13.90	646.86 ± 12.00	756.48 ± 18.225	73.84 ± 52.21
	Formlabs Biomed 50A_1A 48 h	228.00 ± 3.98	below detection level	638.48 ± 26.60	1364.05 ± 72.09	219.73 ± 62.65
	Formlabs Biomed 80A_1A 48 h	198.83 ± 6.85	<13.90	963.32 ± 36.96	1287.7 ± 30.59	73.84 ± 52.21

## Data Availability

The data presented in this study are available on request from the corresponding author due to privacy concerns.

## Abbreviations

The following abbreviations are used in this manuscript:

CSVS	Cardiovascular and thoracic surgeries
WHO	World Health Organization
SMPs	Shape memory polymers
LSs	Lightweight structures
AMCs	(Anti-/meta-) chiral structures
AM	Additive manufacturing
PBF	Powder Bed Fusion
CVTCGI	Cardiovascular and thoracic clamping and gripping instruments
LDH	Lactate dehydrogenase
FISH	Fluorescence in situ hybridization
CLSM	Confocal laser scanning microscope
SEP	Surface energy parameters
